# The Supplementary Motor Area Exerts a Tonic Excitatory Influence on Corticospinal Projections to Phrenic Motoneurons in Awake Humans

**DOI:** 10.1371/journal.pone.0062258

**Published:** 2013-04-16

**Authors:** Louis Laviolette, Marie-Cécile Niérat, Anna L. Hudson, Mathieu Raux, Étienne Allard, Thomas Similowski

**Affiliations:** 1 Université Paris 6, ER10UPMC, Paris, France; 2 Neuroscience Research Australia and University of New South Wales, Sydney, Australia; 3 Assistance Publique-Hôpitaux de Paris, Groupe Hospitalier Pitié-Salpêtrière, Service de Pneumologie et Réanimation Médicale, Paris, France; UCLA, United States of America

## Abstract

**Introduction:**

In humans, cortical mechanisms can interfere with autonomic breathing. Respiratory-related activation of the supplementary motor area (SMA) has been documented during voluntary breathing and in response to inspiratory constraints. The SMA could therefore participate in the increased resting state of the respiratory motor system during wake (i.e. "wakefulness drive to breathe").

**Methods:**

The SMA was conditioned by continuous theta burst magnetic stimulation (cTBS, inhibitory) and 5 Hz conventional rTMS (5 Hz, excitatory). The ensuing effects were described in terms of the diaphragm motor evoked response (DiMEPs) to single-pulse transcranial magnetic stimulation over the motor cortex. DiMEPs were recorded at baseline, and at 3 time-points ("post1", "post2", "post3") up to 15 minutes following conditioning of the SMA.

**Results:**

cTBS reduced the amplitude of DiMEPs from 327.5±159.8 µV at baseline to 243.3±118.7 µV, 217.8±102.9 µV and 240.6±123.9 µV at post 1, post 2 and post 3, respectively (F = 6.341, p = 0.002). 5 Hz conditioning increased the amplitude of DiMEPs from 184.7±96.5 µV at baseline to 270.7±135.4 µV at post 3 (F = 4.844, p = 0.009).

**Conclusions:**

The corticospinal pathway to the diaphragm can be modulated in both directions by conditioning the SMA. This suggests that the baseline respiratory activity of the SMA represents an equipoise from which it is possible to move in either direction. The resting corticofugal outflow from the SMA to phrenic motoneurones that this study evidences could putatively contribute to the wakefulness drive to breathe.

## Introduction

Breathing is the only autonomic function that depends on an extrinsic motor command. Ventilation of the lungs to ensure gas exchange indeed involves contractions of respiratory muscles driven by the motor outflow of spinal motoneurons. In this context, contractions of the diaphragm, the main inspiratory muscle during resting breathing in humans, depend on the firing of phrenic motoneurons. This phrenic activity is the net result of the various inputs received by phrenic motoneurons, including the rhythmic ventilatory drive produced by brainstem central pattern generators responsible for automatic ventilation and its adaptation to the body's metabolic needs via chemosensory feedback [1], [2]. In addition to this bulbospinal input, phrenic motoneurons also receive corticobulbospinal motor inputs from limbic structures [3]–[5] that probably account in part for emotional modulations of breathing [6]–[9]. Higher cortical circuits are a third source of inputs to phrenic motoneurones [10]–[16] that allow voluntary breathing control and interplay between respiration and cortical non-respiratory processes, primarily speech. The respiratory behaviour observed at a given point in time consequently reflects integration of voluntary and involuntary, rhythmic and non-rhythmic central drives, further modulated by various respiratory and non-respiratory afferents [17], [18]; see review in [19].

The importance of this integration is illustrated by the so-called "wakefulness drive to breathe" phenomenon [20]–[23]; review in [19]. During sleep, a decrease in the partial pressure of carbon dioxide in arterial blood induced by hyperventilation (hyperventilation-induced hypocapnia) is associated with apnoea [23], [24]. This phenomenon reflects the "conditional" nature of the respiratory central pattern generator that becomes inactive in the absence of chemical drive to breathe. In contrast, in awake healthy humans, hypocapnia generally fails to induce apnoea [20], [23], [25]. The exact neurophysiological substrates of this phenomenon have not been fully elucidated. The observation of hypocapnia-induced apnoea in an awake patient with locked-in syndrome after bilateral infarction of the ventral pons, and therefore deprived of corticospinal and corticobulbar projections [26], suggests a cortical origin of the "wakefulness drive to breathe". However, it is unknown whether or not and to what extent the putative cortical structures and/or circuits responsible for sustained breathing during hypocapnia exert an influence on normocapnic resting breathing.

Cortical motor neurons projecting onto phrenic motoneurons have been identified in the primary motor cortex (M1) [10], [12], [27], [28] by observation of diaphragm contractions in response to electrical or magnetic stimulation of the brain. Diaphragm responses to transcranial magnetic stimulation over a cortical area anterior to M1 have also been described [29], considered to be evidence of connections between the supplementary motor area (SMA) and phrenic motoneurons [29]. The SMA plays a major role in the planning and execution of movements (review in [30]). It also has a major inhibitory role, particularly in situations involving renouncing a planned motor sequence, stopping this sequence after it has started, or switching to a different sequence [31]–[34]; review in [30]. Electroencephalographic and functional imaging studies indicate that like locomotor movements, voluntary respiratory movements involve the SMA [35]–[39]. There is also evidence that the SMA belongs to cortical circuits engaged in the physiological reaction to inspiratory constraints. When breathing is made experimentally laborious by application of inspiratory resistance, electroencephalographic signs of SMA activation can be observed in the form of slow negativity preceding inspiration in the EEG derivations overlying the vertex [37]–[39]. In particular, a relationship has been reported between SMA activity and unpleasant respiratory sensations (dyspnoea) [37], [38].

The SMA therefore appears to exert an excitatory influence on the production of particular respiratory movements such as voluntary inspirations and constrained inspirations. We hypothezised that the SMA could be a putative source of a resting, "tonic", corticospinal drive to breathe. We therefore set out to test this hypothesis by assuming that, if this were the case, inhibitory conditioning of the SMA by repetitive transcranial magnetic stimulation (rTMS) would result in a depressed response of the diaphragm to stimulation of its primary motor representation (M1dia).

## Materials and Methods

### Subjects

With the approval of the appropriate French ethics and regulatory authorities (*Comité de Protection des Personnes Ile-de-*France VI, Groupe hospitalier Pitié-Salpêtrière, Paris, France), we recruited 12 healthy subjects (5 men) with no history of pulmonary or neuromuscular disease (age: 25±4 years; height: 174.7±11.7 cm; weight: 73.7 ±15.0 kg; mean±SD). They had no previous experience whatsoever with physiology experiments. All volunteers received detailed information and gave their written informed consent.

### Experimental set-up

The subjects were seated in a comfortable chair. EMG recordings were obtained using pairs of surface electrodes (self-adhesive hydrogel, diameter 20 mm, Comepa, St-Denis, France) placed on cleaned and abraded skin. A ground electrode was placed on one acromion.

For the diaphragm, one electrode was placed in the last palpable right intercostal space between the costochondral junction and the midclavicular line, while the other electrode was placed on the overlying rib at a distance of no more than 2 cm. This electrode placement minimizes the risk of contamination of the signal by the electrical activity of extradiaphragmatic muscles coactivated by TMS [40]. The mechanical response of the diaphragm was assessed by changes in abdominal circumference monitored by means of a piezo respiratory belt transducer (ADinstrument/Pneumotrace II, UFI, Morro Bay, CA) attached to an elastic belt placed at the level of the umbilicus. An increase in abdominal circumference in response to TMS was considered to indicate diaphragm contraction.

Surface EMG of the dominant (right side in all subjects) first dorsal interosseous (FDI) was recorded as a non-respiratory control with a belly-belly arrangement and EMG activity of the dominant (right side in all subjects) abductor hallucis (AH) was recorded with a tendon-belly arrangement.

EMG signals were amplified (10000× for diaphragm and 1000× for FDI and AH) and filtered (band-pass 10 Hz – 1 kHz) using a 1902 signal conditioner (Cambridge Electronic Design Ltd., Cambridge, UK), digitized at 10 kHz using a CED Power 1401 MkII data acquisition interface (Cambridge Electronic Design Ltd., Cambridge, UK) and stored on a personal computer for offline analysis using Signal software (Signal 5.00, Cambridge Electronic Design Ltd, Cambridge, UK).

### Transcranial magnetic stimulation (TMS)

A neuronavigation system (eXimia 2.2.0, Nextim Ltd., Helsinki, Finland) was used during all single-pulse TMS (spTMS) and repetitive TMS (rTMS) acquisitions. This system allowed real-time tracking for reliable and precise positioning of the simulation coil over each hot spot, according to individual anatomical MRIs.

#### Motor hot spots

The motor hot spot (M1) was determined for the diaphragm, FDI and AH muscles in a relaxed state (i.e. no pre-stimulus EMG activity observed online) as the spTMS coil position that elicited the largest MEP from the target muscle.

For the diaphragm, DiMEPs were elicited at the end of tidal expiration using either a double-cone coil (90 mm; Magstim Company, Whitland, Wales, UK) positioned perpendicular to the scalp near or over the vertex with induced current flowing in an anterior direction (n = 8) or a figure-of-eight coil (loop diameter of 70 mm; Magstim Company, Whitland, Wales, UK) with the handle pointing to the left (n = 2). The cone coil was used when DiMEPs could not be elicited using the figure-of-eight coil. For each subject, the same coil was used at each visit for all diaphragm spTMS acquisitions.

Both coils were connected to a Magstim Bistim^2^ 200 (Magstim Company, Whitland, Wales, UK), which delivered a monophasic pulse waveform.

The FDI hot spot was stimulated using a figure-of-eight coil connected to a Magstim Bistim^2^ super-rapid stimulator. The coil was placed tangentially over the scalp with the handle pointing backwards with a 45° angle to the mid-sagittal plane. This arrangement induced posteroanterior current flow almost perpendicular to the central sulcus [41].

The AH was stimulated using a figure-of-eight coil connected to a Magstim Bistim^2^ 200. The coil was placed along the sagittal midline with the handle pointing to the right.

#### Motor thresholds

The resting motor threshold (RMT) was defined as the minimum stimulation intensity (% of maximum stimulator output) that elicited an MEP of at least 50 µV for 5 out of 10 successive stimuli [42]. The RMT was determined for the FDI at rest and for the diaphragm at end-expiration.

Active motor threshold (AMT), determined for the FDI and the AH during muscle contraction (20% of maximum voluntary activity, monitored with EMG), corresponded to the stimulation intensity (% of maximum stimulator output) that elicited an MEP ≥ 200 µV for 5 out of 10 successive stimuli.

For RMT and AMT measurements, the stimulator output was decreased by 5% increments until an estimation of the threshold was obtained and then by 1% increments to obtain precise measurement of the threshold. Motor thresholds were measured at each visit.

The RMT of both the FDI and the diaphragm were used to elicit the FDIMEPs and DiMEPs. The AMT of the FDI was used to determine the stimulation intensity of the rTMS protocols and the AMT of the AH was used to locate the SMA [43].

In 3 subjects, the effects of cTBS on RMT and AMT of the diaphragm, FDI and AH were evaluated during a separate session.

#### SMA localization and conditioning

The SMA was identified as being 1 cm anterior to the last point that elicited an MEP from the AH during contraction (20% of maximum voluntary activity) with a stimulation intensity of 120% of the AH AMT [43]. The last point was determined by moving the stimulation coil anteriorly from the AH hot spot over the midsagittal line. Anatomical MRI for each subject was analyzed retrospectively to validate the positioning of stimulation coil over the SMA [44].

The SMA was conditioned by using:

1. an inhibitory theta-burst rTMS protocol, cTBS. Three 50 Hz pulses repeated every 200 ms (i.e. at 5 Hz) continuously for 40 s (for a total of 600 pulses) were delivered at 80% of the AMT of the FDI over the SMA [45].
2. a facilitatory 5 Hz rTMS protocol. Ten second 5-Hz trains, separated by 50 s inter-train, repeated 10 times (for a total of 500 pulses) were delivered over the SMA at 110% of the AMT of the FDI. This facilitatory protocol was selected as it has been previously shown to increase DiMEPS after SMA conditioning [46].

A biphasic waveform stimulator (Magstim Bistim^2^ super-rapid stimulator) with a figure-of-eight coil (loop diameter: 70 mm) was used to deliver the two stimulation protocols. The coil was placed tangentially to the scalp with the handle pointing to the left.

As an increase in FDI EMG activity during SMA conditioning could be due to spread of stimulation over M1, suggestive impending seizure [47], [48], the FDI electromyogram was therefore monitored during rTMS sessions as a precaution.

### Experimental protocol

**Figure 1:**
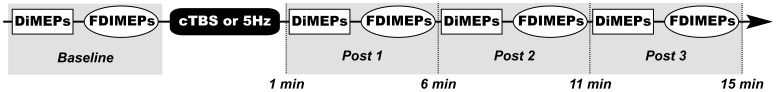
Experimental design. Twenty motor-evoked potentials were recorded for both the diaphragm (DiMEPs) and the first dorsal interosseous (FDIMEPs) at baseline and at three time points (Post 1, Post 2 and Post 3) after the rTMS protocols (cTBS or 5 Hz) over the supplementary motor area (SMA). The time indicates time after the end of rTMS.

The study consisted of 3 visits. The preliminary visit consisted of an anatomical MRI (3D T1-weighted images), while the neurophysiological recordings were performed at the second and third visits. These visits started with RMT assessment for the diaphragm followed by acquisition of 20 DiMEPs at an intensity of 120% of the DIA RMT delivered at the end of tidal expiration. The FDI RMT was then measured, followed by acquisition of 20 FDIMEPs at 120% of the FDI RMT and measurement of the FDI AMT. The AH AMT was then determined, followed by localization of the SMA, over which the rTMS protocol was then administered. DiMEPs and FDIMEPs were measured at 3 time-points following rTMS: 1–5 min (post 1), 6–10 min (post 2) and 11–15 min (post 3) post-rTMS using the same stimulator intensity as at baseline (Figure 1). The rTMS protocols (cTBS and 5 Hz) were administered in a randomized order. An interval of at least 1 week was observed between protocols to avoid carry-over effects.

### Data analysis

The DiMEPs were included in the analysis when they met the following criteria: (1) absence of obvious electrical interference, reflected by clear return of the EMG signal to baseline after the stimulation artefact and before the muscle response; (2) absence of contamination from electrocardiographic signal; (3) concomitant response with abdominal expansion measured by abdominal strain gauge. FDIMEPs for which EMG activity was present before stimulation were excluded from the analysis. For the DiMEPs and FDIMEPs included in the analysis, the level of pre-stimulus EMG was measured as the root mean square (RMS) amplitude over 100 ms prior to stimulation.

MEP amplitudes were measured from peak to peak. All recordings were analysed independently by two observers (LL and MCN) and a third observer resolved any discrepancies (AH). MEP latencies were measured as the time interval between the stimulation pulse and the first excursion of the EMG signal from baseline. For each subject,

MEP amplitude, latency and pre-stimulus EMG were averaged across trials for each muscle and each time-point.

### Statistical analysis

Normal data distribution was checked by Shapiro-Wilk test. As motor threshold intensities (resting motor threshold, RMT and active motor threshold, AMT) presented a non-normal distribution, a Friedman repeated measure analysis of variance followed by Tukey's *post hoc* tests were used to analyze differences between cTBS and 5 Hz conditioning sessions. DiMEP amplitude ( µV and% of baseline), pre-stimulus EMG ( µV) and latencies (ms) had a normal distribution; one-way repeated measure analysis of variance (ANOVA) followed by Holm-Sudak *post hoc* tests were used to test differences between baseline and values at the 3 time-points. FDIMEP amplitude ( µV and% of baseline) and pre-stimulus EMG ( µV) presented a non-normal distribution; a Friedman repeated measure analysis of variance followed by Tukey's *post hoc* tests were used to analyze differences between baseline and values at the 3 time-points. FDIMEPs latencies (ms) were distributed normally and a Friedman repeated measure analysis of variance followed by Tukey's *post hoc* tests were used to analyze differences between baseline and values at the 3 time-points. Effect size was computed according to Cohen [49].

Values of p<0.05 were considered statistically significant. Data are expressed as both percentage of baseline MEP, with baseline corresponding to 100%, and absolute values. Values are shown as mean±SD (DiMEPs) or median [Q1–Q3] (FDIMEPs) depending on the distribution. Statistical analyses were performed using SPSS v11.5 for Windows (SPSS Inc., Chicago, IL, USA).

## Results

Two of the 12 subjects withdrew their consent (one before the initial experimental session without giving a reason and the other during the first session because of discomfort during spTMS). A third subject only attended the preliminary visit and the first experimental visit (cTBS protocol) and subsequently withdrew from the study because of an unrelated medical reason. The results therefore concern 10 subjects for cTBS data and 9 subjects for 5 Hz data.

### Safety

The subjects did not report any side effects during or after any of the experimental sessions. FDI monitoring during rTMS never revealed EMG patterns suggestive of spread of the rTMS stimulation over SMA to the primary motor cortex.

### Stimulation localization

In all the subjects, extensive motor mapping of the diaphragm response to spTMS identified a M1 hotspot in the vertex region, with neuronavigation coordinates fully compatible with previously provided descriptions [15], [28], [46]. Also, in all the subjects, the SMA hot spot was located approximately 3 cm anteriorly to the vertex, which corresponds to data previously reported in several other studies [43], [46], [50] (See figure 1. in [46]). Post-hoc MRI localization according to Picard and Strick [44] consistently confirmed that the SMA hot spots corresponded to the anatomical SMA region.

### Resting and active motor thresholds

RMT of the diaphragm and FDI and AMT of FDI and AH are shown in Table 1. They did not vary significantly between visits. In another 3 subjects, we tested whether the conditioning protocols over the SMA altered the DIA and FDI RMT and the FDI and AH AMT. No changes were observed throughout the session.

**Table 1:** Resting (RMT) and active (AMT) motor thresholds for the diaphragm, first dorsal interosseous (FDI) and abductor hallucis (AH).

	cTBS				5 Hz			
	*RMT*		*AMT*		*RMT*		*AMT*	
*Diaphragm*								
Figure-of-8 coil	78	[74]–[80]			72	[72–72]		
*(n = 3 for cTBS and n = 2 for 5* *Hz)*								
Cone coil	60	[50]–[67]			55	[46]–[72]		
*(n = 7)*								
*FDI*	54	[52]–[64]	46	[40]–[51]	56	[49]–[67]	44	[36]–[56]
*AH*			63	[51]–[84]			67	[50]–[86]

Values shown are% of maximum stimulator output and expressed as median [range]. Definition of abbreviations: FDI = first dorsal interosseous, AH = abductor hallucis.

### Diaphragm corticospinal excitability following SMA conditioning

**Figure 2:**
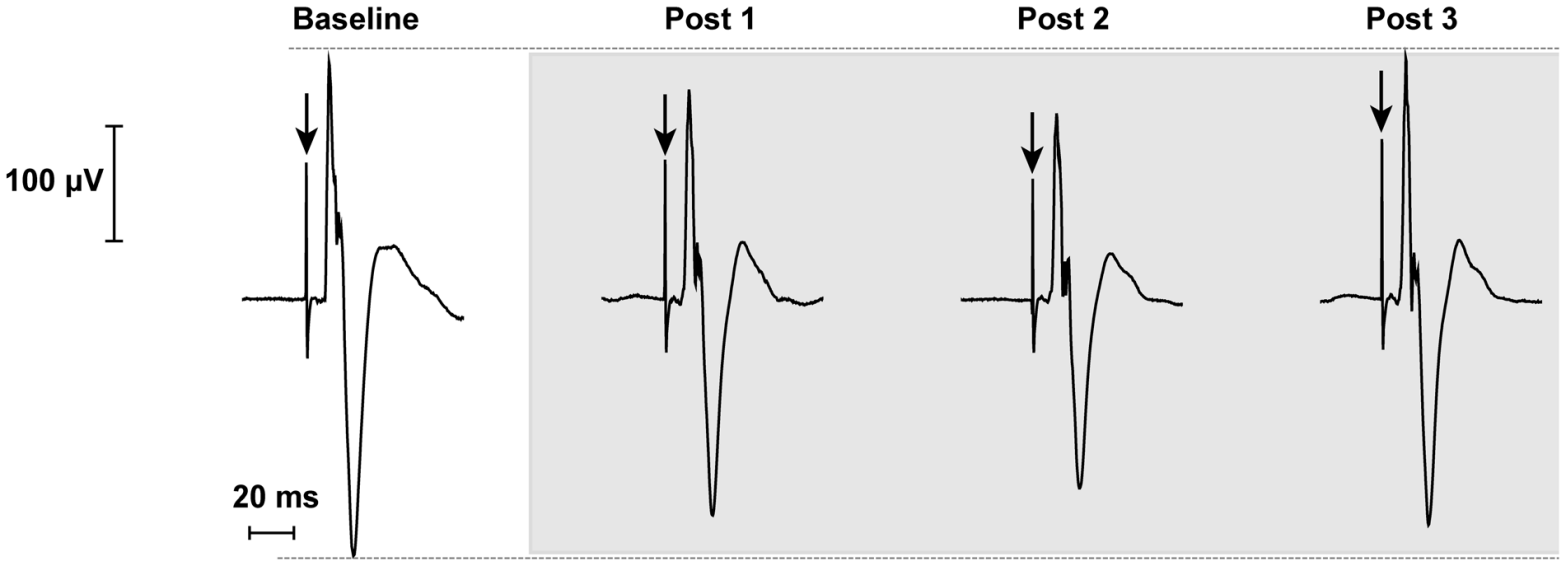
Average waveform of diaphragm motor-evoked potentials (DiMEPs) for a single subject evoked by single-pulse TMS over the motor cortex at baseline and at 3 time-points following the inhibitory protocol (cTBS) over the SMA. Arrows indicate the time of stimulation.

**Figure 3:**
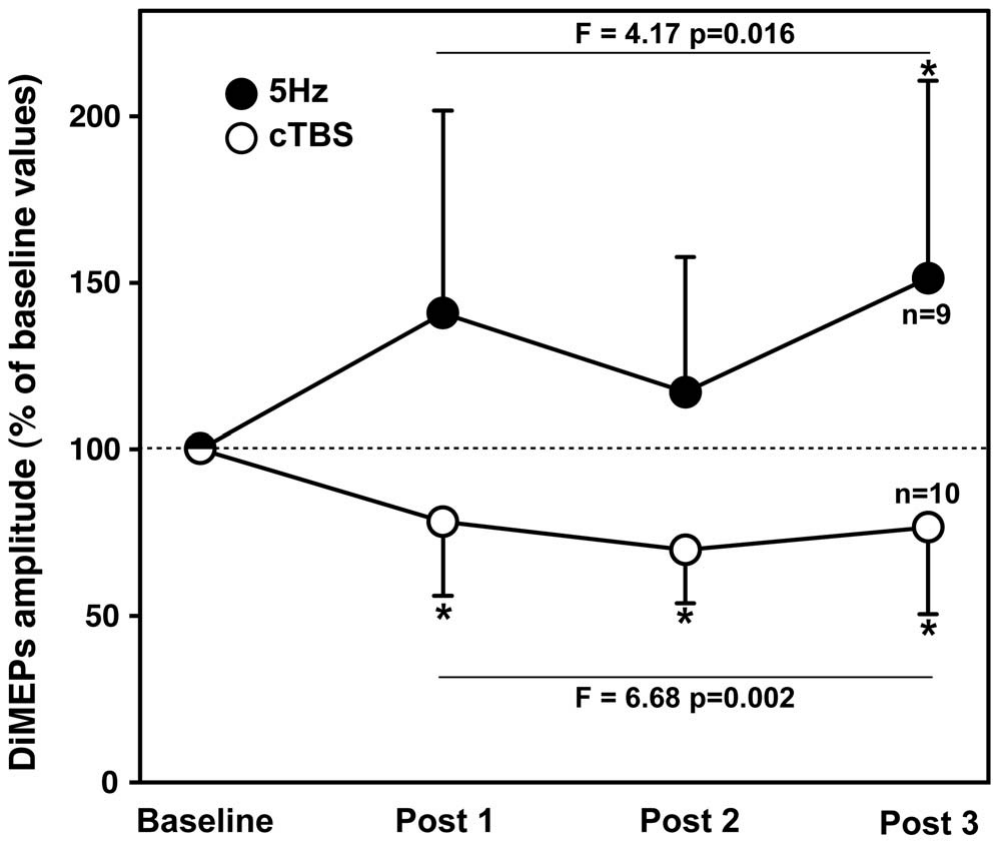
Average amplitude of diaphragm motor-evoked potentials (DiMEPs) for all subjects evoked at baseline and at 3 time-points following inhibitory (cTBS, white circles) and excitatory (5 Hz, black circles) conditioning of the SMA. Values are expressed as% of baseline and as mean±SD. * = p<0.05 vs. baseline following *post hoc* tests.

Following inhibitory conditioning (cTBS), a significant decrease in DiMEPs amplitude (F = 6.341, p = 0.002 when expressed in µV) was observed from baseline to post 1, post 2 and post 3 (see Figure 2, Figure 3 and Table 2). These decreases corresponded to moderate effect-size values [49] of −0.53, −0.69 and −0.54 for post 1, post 2 and post 3, respectively. The decrease in DiMEP amplitude was observed in 80%, 100% and 70% of subjects at post 1, post 2 and post 3 time-points, respectively.

**Table 2:** Amplitude, latencies and 100 ms pre-stimulus RMS EMG of diaphragm motor-evoked potentials (DiMEPs) before/after SMA inhibitory (cTBS) and facilitatory (5 Hz) conditioning protocols.

	cTBS									5 Hz								
	Amplitude ( µV)			Latencies (ms)			Pre-stimulus (100 ms) EMG ( µV)			Amplitude ( µV)			Latencies (ms)			*Pre-stimulus (100* *ms) EMG (* µ*V)*		
*Baseline*	327.5	±	159.8†	15.9	±	1.7	3.1	±	1.1	184.7	±	96.5†	16.1	±	1. 6	3.3	±	0.7
*Post 1*	243.3	±	118.7*	15.9	±	1.7	3.4	±	1.2	244.9	±	111.3	16.2	±	1.4	3.6	±	1.1
*Post 2*	217.8	±	102.9*	16.1	±	1.7	2.8	±	0.5	207.5	±	99.9	16.4	±	1.1	3.5	±	1.2
*Post 3*	240.6	±	123.9*	16.1	±	1.6	2.9	±	0.5	270.7	±	135.4*	16.1	±	1.6	3.6	±	0.6

DiMEP values had a normal distribution and are therefore presented as mean±SD. * = p<0.05 vs. baseline values following *post hoc* tests. † = p<0.05 between cTBS and 5 Hz conditions. The baseline values were statistically different (F = 5.39, p<0.05). This difference was entirely accounted for by the one subject who dropped out of the study before participating in the 5 Hz part of the experiment. When this subject was removed from the analysis, the difference between baselines disappeared, but the cTBS-related inhibition did persist (F = 4.508, p<0.012).

Excitatory conditioning of the SMA (5 Hz) increased DiMEP amplitude (F = 4.844, p = 0.009) compared to baseline, at post 3 only (see Figure 3 and Table 2). This increase corresponded to a large effect-size of 0.89 [49]. The increase in DiMEP amplitude was observed in 89% of subjects for the post 3 time-points.

Pre-stimulus EMG for DiMEPs was similar at all time-points for both the cTBS and the 5 Hz conditioning protocols (see Table 2). DiMEP latency was not affected by the rTMS protocols.

### FDI corticospinal excitability following SMA conditioning

Neither inhibitory (cTBS) nor excitatory (5 Hz) conditioning protocols significantly altered FDIMEP amplitude. (χ2 = 4.2, df = 3, p = 0.241 for cTBS and χ2 = 3.400, df = 3, p = 0.334 for 5 Hz) (Table 3).

**Table 3:** Amplitude, latencies and 100 ms pre-stimulus RMS EMG of first dorsal interosseous motor-evoked potentials (FDIMEPs) before/after SMA inhibitory (cTBS) and facilitatory (5 Hz) conditioning protocols.

	cTBS			5 Hz		
	*Amplitude ( µV)*	*Latencies (ms)*	*Pre-stimulus (100 ms) EMG ( µV)*	*Amplitude ( µV)*	*Latencies (ms)*	*Pre-stimulus (100* *ms) EMG (* µ*V)*
*Baseline*	899.7 [525.8–1582.0]	23.4 [22.0–25.0]	9.8 [8.8–11.3]	1060.7 [704.1–1603.5]	23.1 [21.5–24.3]	9.4 [8.4–11.0]
*Post 1*	495.3 [273.0–1899.0]	23.4 [21.4–24.8]	9.9 [9.2–11.3]	1278.6 [788.7–2227.6]	23.2 [21.5–24.4]	10.2 [9.4–11.9]
*Post 2*	461.6 [149.1–1431.0]	23.1 [21.4–24.6]	10.6 [9.2–11.4]	1595.6 [699.2–2411.1]	23.3 [21.7–24.1]	9.9 [9.5–12.0]*
*Post 3*	821.9 [364.1–1899.7]	23.4 [21.5–24.9]	10.8 [9.3–11.5]	1245.9 [787.3–2323.7]	22.7 [21.8–24.0]	10.9 [9.6–12.0]*

FDIMEP values had a non-normal distribution and are therefore presented as median and [Q1–Q3]. * = p<0.05 vs. baseline values following *post hoc* tests.

Pre-stimulus EMG for FDIMEPs remained unchanged at all time-points for the inhibitory (cTBS) protocol, but was higher at post 2 and post 3 compared to baseline (χ2 = 21.4, df = 3, p<0,001) for the excitatory protocol (5 Hz). FDIMEP latency was not affected by the rTMS protocols.

## Discussion

This study shows that conditioning the SMA with an inhibitory rTMS paradigm depresses the response of the diaphragm to the corticospinal inputs induced by spTMS over M1dia. In addition, it confirms that conditioning the SMA with an excitatory protocol (5 Hz stimulation) enhances the response of the diaphragm to spTMS [46]. These results corroborate the demonstration of a functional connectivity between the SMA and M1dia [46] and complement the description of this connectivity. The present results also suggest that the SMA exerts a "tonic" excitatory influence on the corticospinal drive to phrenic motoneurons during normocapnic resting breathing in healthy, awake humans. This baseline respiratory activity of the SMA probably represents an equipoise from which it is easy to move in either direction (facilitation or inhibition). This might have a particular relevance to respiratory control during speech.

### Methodological considerations

#### EMG recordings

Surface recordings of diaphragm EMG can be contaminated by the activity of adjacent muscles. To limit this possible interpretation bias, we included abdominal expansion in response to spTMS —indicative of an actual diaphragm contraction—as a quality criterion to analyse DiMEPs and we positioned the chest surface electrodes according to a contamination-minimised montage [40].

Underlying contractions enhance motoneuron responsiveness to TMS and increase the amplitude of MEPs of either voluntary origin [51], [52] or, in the case of the diaphragm, driven by automatic breathing control [53], [54]. In our subjects, spTMS was delivered at the end of tidal expiration, when the inspiratory bulbospinal drive to the phrenic motoneurons is absent or minimal [55]. The diaphragm hotspot and RMT were verified during each visit and were reproducible. In addition, the pre-stimulus RMS amplitudes for a given subject were stable during the sessions and reproducible between sessions. We are therefore confident that our recording technique minimized the impact of confounding factors on the results.

#### Conditioning stimulation of the SMA

The SMA hot spot in our subjects was located according to a previously described methodology and its position validated by *post hoc* MRI analysis (see results). We are therefore confident that conditioning stimulations actually reached the SMA, and, at any rate, the supplementary motor complex (SMC). We are also confident that spread of the conditioning stimulation during the experimental sessions was minimal based on continuous use of MRI-guided neuronavigation for coil positioning. Moreover, a spread to M1 was unlikely to have occurred: low stimulation intensities and use of a focal coil should have been responsible for a rapid decay of the magnetic field outside the targeted spot. This is supported by the lack of FDI EMG activity during SMA conditioning. For greater safety [47], [48], the intensity of conditioning stimulations was calibrated according to the FDI AMT (80% and 110% for the cTBS and 5 Hz protocols, respectively) and not according to the diaphragm AMT, which is systematically higher [56]–[58]. Of notice, we chose to monitor FDI activity as an indicator of the spread toward M1 rather than the AH activity. Indeed, even though the AH M1 representation is closer to the site of SMA stimulation, the FDI threshold is lower than that of the AH.

A trend in FDIMEP response was observed after cTBS and 5 Hz conditioning that resembled the diaphragm responses (decreased MEP in response to cTBS, increased MEP in response to 5 Hz). These trends are in line with previous results [46]. However, these results did not reach the limit of statistical significance, possibly because FDIMEP amplitudes were much more variable between subjects than DiMEP amplitudes. This may be the result of insufficient control of FDI muscle activity during the experiments [59]–[62]. Small but significant increases in FDI pre-stimulus EMG were indeed observed between MEPs evoked at baseline and at two time-points following 5 Hz stimulation (Table 3).

### Neurophysiological considerations

#### Connections between the SMA and phrenic motoneurons

**Figure 4:**
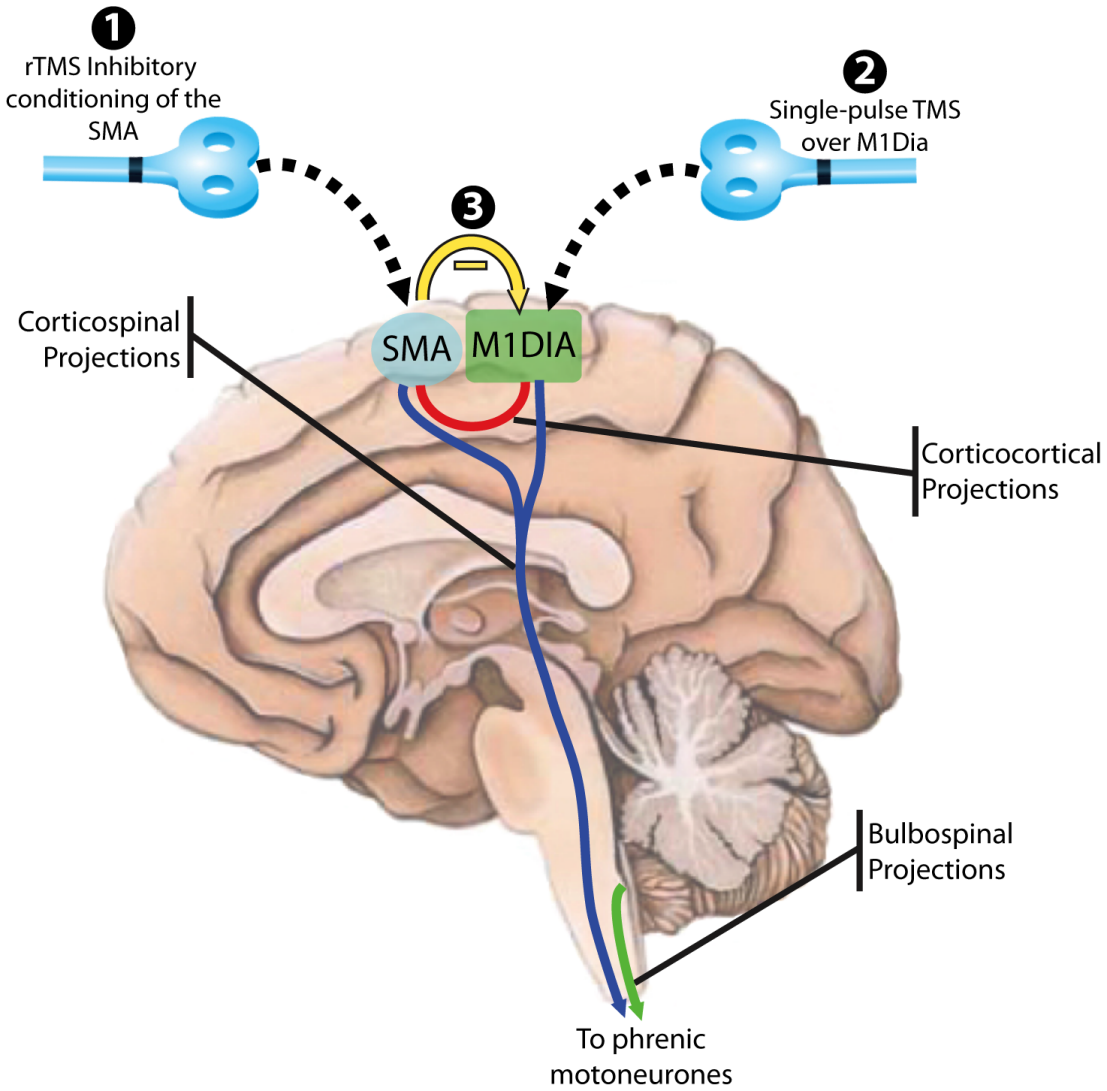
Schematic representations of descending projections to the phrenic motoneurones and corticocortical projections between the SMA and diaphragm primary motor representation (M1Dia). We showed that inhibitory conditioning of the SMA by repetitive transcranial magnetic stimulation (rTMS, “1”) results in a depressed response of the diaphragm to stimulation of M1dia (“2”). We hypothesise that this is due to inhibition of the corticocortical connections between the SMA and MIdia (“3”) and this suggests that there is a resting facilitatory tonic projection between these areas (shown in red). There are other descending pathways to the phrenic motoneurones that are not shown for clarity (i.e. from the limbic cortex).

Sharshar et al. [29] established the likely existence of SMA-phrenic connections by describing DiMEPs in response to TMS applied over two distinct cortical spots, one located at the vertex, the other 3 cm anteriorly. They argued that the vertex spot corresponded to the diaphragm representation within the primary motor cortex (M1dia) and that the more anterior spot corresponded to the SMA. The two cortical areas from which diaphragm responses could be elicited exhibited significant differences in terms of their intracortical inhibitory/excitatory balance and their facilitatory output during voluntary inspiratory efforts. The authors concluded that the SMA likely exerted a predominantly excitatory effect on phrenic motoneurons [29]. They also postulated that they had described a direct SMA-phrenic projection rather than a SMA-M1 relay to the phrenic motoneurons. The main argument supporting this contention was the short latency of the diaphragm response to SMA stimulation and the lack of notable difference between the MEP latencies at the two stimulation spots (16.4±2.7 ms vs. 16.7±2.4 ms under relaxed conditions). However, these findings do not formally rule out a SMA-M1 relay to the phrenic motoneurons that would resemble known functional SMA-M1 connections described for non-respiratory muscles [43], [50], [63]–[67]. Consequently, our observations could reflect modulation of either a direct corticophrenic pathway arising in the SMA or an indirect pathway featuring intracortical SMA-M1dia connections (Figure 4). In the present study, as in a previous study [46], we observed that 5 Hz conditioning stimulation facilitated the diaphragm response to spTMS. DiMEP amplitudes increased, but no latency effect was observed. This pattern of MEP changes is suggestive of cortical facilitation, as opposed to spinal facilitation that involves combined spatial and temporal motor unit recruitment and is associated with both increased amplitude and decreased latency of the facilitated MEPs [68]; see detailed discussion in [54]. In addition, available data indicate that rTMS delivered over the primary motor cortex and over the premotor cortex does not modify spinal motoneuron excitability as assessed by the H-reflex [43], [45], [69], [70]; review in [71]. Other data indicate that rTMS conditioning protocols exert their effects via cortico-cortical mechanisms [45], [72]–[75]. All in all, we therefore propose that the DiMEP changes observed after SMA conditioning in our subjects resulted not from modulation of direct SMA-phrenic projections, but from modulation of SMA-M1-phrenic connections. However, formal proof of this contention is currently lacking. This proof could be obtained by studying the effects of SMA conditioning on the diaphragmatic response to paired-pulse TMS [16]. It is also important to consider that the SMA most likely operates as a node from a larger motor-control network of subcortical structures, including the basal ganglia, thalamus, cerebellum and pons [30], [76]–[78].

#### Influence of the SMA on the M1Dia-phrenic projections

The fact that inhibitory conditioning of the SMA resulted in a decreased diaphragm response to TMS implies the existence of a resting corticofugal outflow from the SMA to phrenic motoneurons. It is indeed unlikely that an inhibitory effect following an inhibitory conditioning could be observed in the absence of a prior tonic facilitatory drive.

Our observations contrast with those of a previous study by Raux et al. [46], in which facilitatory conditioning of the SMA (5 Hz rTMS) enhanced the response of the diaphragm to spTMS over M1dia, but an inhibitory rTMS protocol (i.e. 1 Hz rTMS) failed to induce any reduction in the amplitude of DiMEPs. The difference between the two studies is probably related to differences in inhibitory efficiency between the two conditioning paradigms, namely 1 Hz rTMS vs. cTBS [45], [79]. Our observations are somewhat reminiscent of the demonstration of effective facilitatory connectivity between the SMA and M1 in the absence of movement [67]. They are also compatible with the observation that mechanical ventilation, which applies a positive pressure to the airway and unloads the respiratory muscles from their ventilatory task, depresses the excitability of the corticophrenic pathway in awake humans [80]. Sharshar et al [80] observed that the institution of isocapnic mechanical ventilation through a face mask in normal individuals reduced the amplitude of DiMEPs. As paired TMS stimulation concomitantly showed that mechanical ventilation was associated with an enhanced response to paired TMS at facilitatory interstimulation intervals. The authors concluded that the observed inhibition occurred at a cortical site. As no such changes were observed for the quadriceps, they concluded that the observed phenomenon was specific to the diaphragm. Sharshar et al. [80] postulated that the SMA could be among the cortical regions involved in breathing control, as inhibition of the SMA would explain their results. However, it is known that inspiratory efforts giving rise to negative airway pressure —the opposite of what occurs during mechanical ventilation— evoke cortical potentials indicating respiratory-related brain activation [81]. Source dipole analysis of the early components of these potentials demonstrated involvement of the SMA [82]. It can therefore be hypothesized that afferent feedback from the respiratory system provides permanent excitatory input to the SMA, which would in turn correspond to the excitatory efferent outflow documented in the present study. By silencing this afferent feedback, mechanical ventilation would therefore result in the intracortical inhibition described by Sharshar et al. [80].

#### Implications for breathing control and conclusions

If, as our data suggest, the SMA does exert a resting facilitatory influence on phrenic motoneurons, and if this influence depends on SMA-M1 intracortical connections, then the sleep-related loss of intracortical connectivity [83] could reasonably be hypothetized to contribute to the sleep-related reduction in ventilatory activity and the reduced diaphragm response to spTMS during sleep [54]. We may therefore have identified one of the neurophysiological substrates of the increased resting state of the respiratory motor system that characterizes wakefulness (the the so-called "wakefulness drive to breathe"). While we acknowledge that our observations do not prove that the SMA participates to the wakefulness drive to breathe, we however think that they provide a sufficient rationale to devise specific experiments with this objective. For example, one would expect that inhibiting the SMA before inducing hypocapnia in awake subjects would result in an increased occurrence of post-hyperventilation apneas.

This study adds to the current body of knowledge suggesting that the SMA plays a significant role in breathing control. Given the role of the SMA in movement preparation and inhibition (review in [30]), it can be postulated that it plays a fundamental role in the production of the voluntary inspirations that intersperse speech and in the inhibition of prepared speech-related breaths during adaptations to conversational environment [84]. Our demonstration that the SMA can be both facilitated (5 Hz protocol) and inhibited (cTBS protocol) in the present study goes in this direction. The SMA could also participate in inhibition of the activity of the central pattern generators [85] that is essential to ensure that speech is not disrupted by "metabolic" breaths. In this regard, the SMA does belong to a cortico-subcortical network that is activated during voluntary breath-holding [86]. Finally, the SMA is involved in the physiological response induced by experimental respiratory constraints [37]. Such constraints elicit unpleasant respiratory sensations [37]. The present observations therefore provide a preliminary rationale to study rTMS as a method to alleviate certain forms of dyspnea, in the same manner as it is used to relieve certain forms of pain [87].

## Acknowledgments

We would like to thank the Centre de Recherche de l’Institut du Cerveau et de la Moelle épiniere (CRICM), the Plateforme de neurophysiologie de l’Institut du Cerveau et de la Moelle épinière (ICM) for their help in conducting this study and Laurence Jacquenod, TICEMED, Université Paris 6 Pierre et Marie Curie, Paris, France for her help with figure 4.
